# Transactions of the Illinois State Medical Society

**Published:** 1860-12

**Authors:** 


☞ The Transactions of the Illinois State Medical Society have at last come into being, after a lingering and tedious labor, from among the viscera of the *Examiner*. We have not yet had time to give the pamphlet a full reading, but it does strike us as singular that after such a prolonged gestation, the product shows either "arrest of development," or evisceration. The copies we have seen have omitted eight pages from the body of the report on Surgery. Comment is unneccessary. We shall read the balance at our leisure.

